# Response of Plants to Touch Stress at Morphological, Physiological and Molecular Levels

**DOI:** 10.3390/ijms262211120

**Published:** 2025-11-17

**Authors:** Agata Jędrzejuk, Natalia Kuźma

**Affiliations:** Institute of Horticultural Sciences, Department of Environmental Protection and Dendrology, Warsaw University of Life Sciences, Nowoursynowska 159, 02-787 Warsaw, Poland; natalia_kuzma@sggw.edu.pl

**Keywords:** thigmomorphogenesis, plant response to stress, TCH genes, plant hormones, defending enzymes, oxygenases

## Abstract

Thigmomorphogenesis denotes a suite of anatomical, physiological, biochemical, biophysical, and molecular responses of plants to mechanical stimulation. This phenomenon is evolutionarily conserved among diverse plant lineages; however, the magnitude and character of the response are strongly determined by both the frequency and intensity of the applied stimulus. In angiosperms, thigmomorphogenetic reactions typically occur gradually, reflecting a complex interplay of morphological alterations, biochemical adjustments, and genetic reprogramming. In dicotyledonous plants, thigmomorphogenesis is commonly expressed as a reduction in leaf blade surface area, shortening of petioles, decreased plant height, radial thickening of stems, and modifications in root system architecture. In monocotyledons, in turn, mechanical stress frequently results in stem rupture below the inflorescence, with concomitant shortening and increased flexibility of younger internodes. These specific traits can be explained by structural features of monocot secondary walls as well as by the absence of vascular cambium and lateral meristems. Mechanical stimulation has been shown to initiate a cascade of responses across multiple levels of plant organization. The earliest events involve activation of mechanoresponsive genes (e.g., TCH family), followed by enzymatic activation, biochemical shifts, and downstream physiological and molecular adjustments. Importantly, recent findings indicate that prolonged mechanical stress may significantly suppress auxin biosynthesis, while leaving auxin transport processes unaffected. Moreover, strong interdependencies have been identified between thigmostimulation, gibberellin biosynthesis, and flowering intensity, as well as between mechanical stress and signaling pathways of other phytohormones, including abscisic acid, jasmonic acid, and ethylene. At the molecular scale, studies have demonstrated a robust correlation between the expression of specific calmodulin isoforms and the *GH3.1* gene, suggesting a mechanistic link between mechanosensing, hormone homeostasis, and regulatory feedback loops. The present study consolidates current knowledge and integrates novel findings, emphasizing both morphological and cellular dimensions of thigmomorphogenesis. In particular, it provides evidence that mechanical stress constitutes a critical modulator of hormonal balance, thereby shaping plant growth, development, and adaptive potential.

## 1. Introduction

Thigmomorphogenesis is a phenomenon that remains not fully understood, and the available literature on this topic has developed mostly in recent decades. The phenomenon was first described in 1973 by Jaffe [1]; however, similar observations have been made for thousands of years in cereals, where the practice was known as mugifumi [2]. In Greek, thigmo means “I touch,” and morphogenesis refers to the generation of form; thus, thigmomorphogenesis refers to touch-induced morphological changes [3].

Plant species exhibit diverse morphological responses to prolonged mechanical stress [4]. Over billions of years, plants have evolved specialized mechanisms to counteract abiotic stressors that modulate their growth. In response to environmental stress, plants activate molecular, physiological, and morphological processes, including the release of Ca^2+^ ions, alterations in phytohormone synthesis, changes in reactive oxygen species (ROS) dynamics [5,6,7,8,9], hormonal modulation, altered gene expression, and cytoskeletal reorganization [10,11,12]. Thigmomorphogenesis is typically characterized by a reduction in leaf area, petiole length, and plant height, accompanied by increased stem thickness and modifications in root architecture [13,14]. Many plant species have retained their capacity for a rapid response to mechanical stimuli, improving acclimation under unstable environmental conditions [15]. Growth modification represents an adaptive strategy that enables plants to withstand additional mechanical stressors [16]. Morphogenetic alterations induced by tactile stimulation are often associated with enhanced production of strengthening tissues in herbaceous plants [10,17,18]. However, in certain species, such stimuli can also result in increased tissue flexibility [4,17,19,20,21]. For instance, in sunflower (Helianthus annuus), increased stem stiffness and strength have been observed following mechanical stimulation [22].

Although thigmomorphogenesis is a widely observed phenomenon across the plant kingdom, responses differ markedly between woody and herbaceous species. In woody plants, reactions to mechanical stimuli primarily involve alterations in wood structure and secondary stem growth, leading to architectural modifications at the whole plant level [15,23,24,25]. Such responses may include increased periderm thickness as an adaptation to external forces such as wind. Growth may become more compact, with fewer lateral shoots, reduced leaf or needle surface area, and denser foliage [10,17,19,26,27]. Flexure wood, formed as a response to external forces, is a specialized type of wood characterized by increased radial growth in the direction of mechanical stress [28]. This process can result in thickened cell walls, altered cellulose microfibril orientation, reduced diameter and length of tracheary elements, and a lower vessel-to-fiber ratio in angiosperms [28]. In addition to flexure wood, reaction wood may also form, featuring modified secondary cell wall layers in which the lignified layers are replaced by a thick, unlignified gelatinous layer [29].

In herbaceous plants, responses to mechanical stimuli generally involve direct growth modifications, such as inhibited shoot elongation, increased stem diameter, and alterations in leaf structure (thickness and area) [14,15,16,30,31,32]. Plant responses to mechanical stress represent a crucial component of adaptation to environmental variability. The speed at which plants respond to mechanical stimuli can be remarkably rapid, with initial biochemical changes occurring within seconds of stress exposure [21]. Research on response dynamics can be broadly divided into short-term and long-term studies. Long-term investigations provide insights into growth responses occurring over days or months. Both short- and long-term adaptations to mechanical stress are essential for plant survival in dynamic environments.

## 2. Plant Response on the Morphological and Anatomical Level

**Figure 1:**
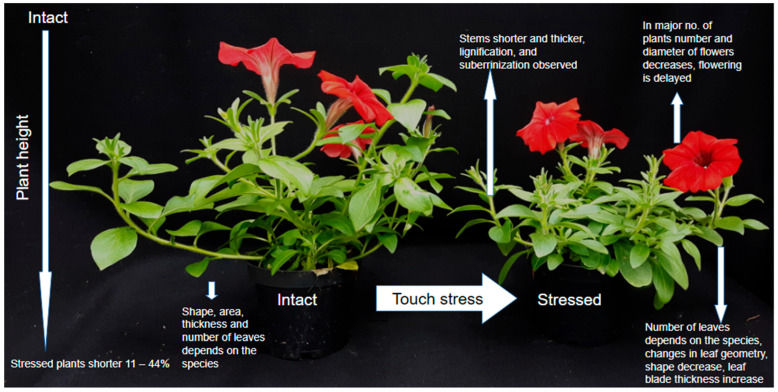
Morphological Change Schematic Comparison.

Alteration of plant growth patterns in response to mechanical stress has been the subject of numerous studies on various plant species [1,21,33], including ornamental plants [13,20,30,34,35] and trees [15,16,30,36]. One of the most common thigmomorphogenetic responses is the inhibition of stem elongation accompanied by an increase in stem diameter [14,16,20,30,35,37,38], Figure 1. Mechanical stress significantly reduces plant growth in several species: *Helianthus annuus* by 14%, *Euphorbia pulcherrima* by 11%, *Scaevola aemula* by 39%, *Ocimum basilicum* by 31%, *Aster dumosus* by 25%, *Serianthes nelsonii* by 17%, *Pelargonium* sp. by 21%, *Calibrachoa* sp. by 24%, and *Petunia* × *atkinsiana* ‘Pegasus ^®^ Velvet Picotee’ by 43.5% [13,30,39,40]. Plants subjected to mechanical stimulation are typically shorter and develop thicker stems [41].

According to Jędrzejuk et al. [34], the processes of lignification and suberization occurring in the cells of mechanically stimulated plants also contribute to growth inhibition. Cellular growth and division depend on the balance between turgor pressure and the mechanical resistance of the cell wall [27]. Alterations in plant geometry caused by mechanical pressure are a key determinant of cell remobilization, given the prominent role of microtubules in cell division. It is worth noting that, in plant cells, locomotion does not occur, and cell shape is constrained by the rigid cell wall [42]. Mechanical stress affects not only the overall plant architecture but also leaf morphology [30,43,44]. In an experiment on tomatoes, repeated touching of the shoot apical meristem resulted in significant reductions in leaf area, leaf number, and dry mass [43]. In the study by Jędrzejuk et al. [27], mechanical stress did not affect leaf area in *Petunia* × *atkinsiana* ‘Dark Red’ [30]. However, by the end of the experiment, another tested cultivar, ‘Pegasus ^®^ Velvet Picotee’, responded differently, exhibiting a reduced leaf area compared to control plants. Studies on Urtica dioica demonstrated directional changes in leaf geometry in response to mechanical stimuli [44]. In experiments by Anten et al. (2010), Plantago major exhibited elongation and thinning of leaf blades as well as increased petiole length following tactile stimulation [43,45]. Conversely, wind exposure in the same study produced effects opposite to brushing. Cipollini (1997) reported increased leaf blade thickness in Phaseolus vulgaris subjected to mechanical stress [44,46]. According to Anten et al. (2010), these differences in adaptive responses are related to the type of mechanical stimulus and/or the function of the affected organ [43,45].

Stress is a major factor considered to disrupt the flowering process [47,48,49,50,51,52,53]. Acceleration or inhibition of stress-induced flowering represents an adaptive response to environmental cues [30,54]. The direction of this response depends on the stimulus type, plant species, and even cultivar [27,45,53]. According to Telewski (2021), thigmomorphogenesis generally reduces the number of flowers and delays flowering [4]. These findings were previously confirmed in studies by Braam [14], Chehab et al. [18], Graham and Wheeler [54], and Jędrzejuk et al. [27]. In the latter study, *Petunia* × *atkinsiana* ‘Pegasus ^®^ Velvet Picotee’ produced on average 14 fewer flowers per plant under mechanical stress. However, another cultivar exposed to 80 touches per day exhibited an increase of 11 flowers per plant. In both cultivars, no significant effect of mechanical stress on flower diameter was observed. In an experiment on Capsicum annuum ‘California Wonder’, a reduction of 10 flowers per plant and a significant delay in flowering were reported compared with unstressed controls [54]. Mechanical stress also influences root architecture [25,27,30,55,56]. Darwin was the first to observe mechanical effects on root growth [57]. He attached a small piece of mica to one side of a pea root tip, causing the root to bend away from the point of contact. Primary roots play a crucial role in anchoring small plants and young trees [57,58,59]. The main strategy for counteracting environmental stress involves structural and developmental adaptation of the root system disturbances in accordance of touch, by development of lateral roots. However, studies by Reubens et al. (2009) demonstrated that the biomass distribution between roots and shoots depends on the species [59]. In herbaceous plants, an increase in the number and thickness of lateral roots was observed following regular bending of aerial parts [60,61,62].

The findings presented above highlight the complexity of plant adaptive responses to mechanical stress and their dependence on multiple factors. The earliest discernible effect of mechanical stress on plant growth is growth inhibition [3]. Growth cessation is typically observed between three and six minutes after mechanical stimulus application [3,21,63,64,65,66]. The duration of growth inhibition differs by species. In tomatoes growth ceases ca. one hour after the touch [21]. Telewski et al. (2021) claims that after touch stress, normal growth resumes after approximately 30–60 min [4], that was previously proved [67,68,69]. Notably, compensatory growth does not occur to offset the period of arrested growth [70,71].

Regarding plant stem diameter, following growth cessation, an increase in radial expansion rate is observed as a consequence of mechanical bending [3]. In poplar, the radial growth of young stems continues two to three days before decreasing and returning to pre-bending levels. According to Telewski et al. (2021), radial expansion of the stem occurs between six and twenty-four hours after the stressor is applied [4]. Cells adjacent to tissue layers directly pressurized by the stressor may be several times thicker than other cells [72].

## 3. Plant Response at the Hormonal and Enzymatic Level

**Figure 2:**
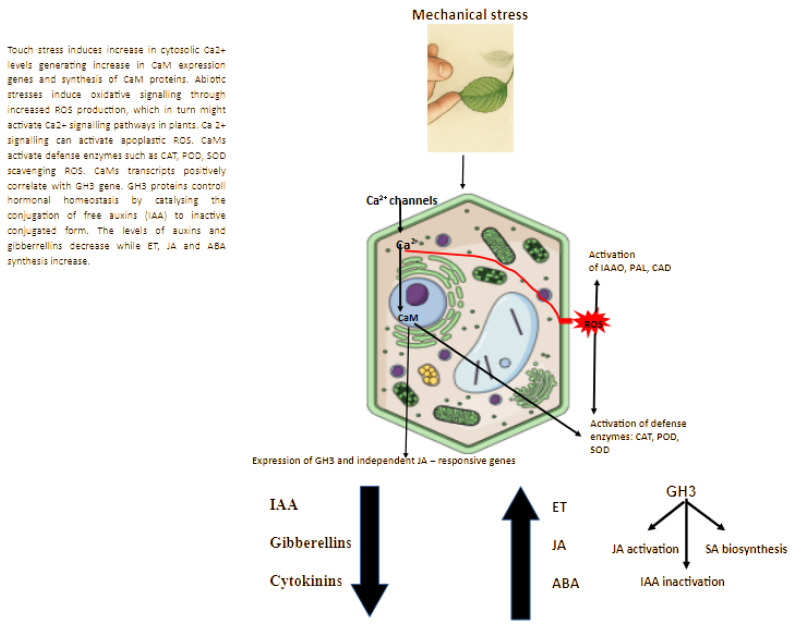
Thigmomorphogenesis Pathway Overview.

Plants respond to mechanical stress through a complex network of hormonal and enzymatic signaling pathways (Figure 2). According to Wang et al. (2024) [71,73] nearly all phytohormones actively participate in modulating plant responses to touch stress [9,40,46,64,65,74,75,76].

### 3.1. Phytohormones

**Auxins**

Touch stress has been shown to reduce IAA levels in several plant species, such as Plantago major, Solanum lycopersicum, Cyclanthera brachybotrys, Phaseolus vulgaris, and Solanum tuberosum [76,77,78,79,80]. In some plants, i.e., petunia, a decrease in IAA concentration was observed 30 days after the onset of stress [34]. Furthermore, mechanical stress did not appear to influence auxin transport in stems or its content in roots, regardless of treatment type or stress duration. This experiment clearly demonstrated that mechanical stimulation affected auxin biosynthesis in the shoot apical meristem (SAM) only after prolonged exposure, approximately 30 days after the onset of stress. There is limited information comparing IAA content between the SAM and root apical meristem (RAM). In studies conducted by Jędrzejuk et al. (2023), a significantly lower IAA concentration was observed in the RAM than in the SAM of the investigated plants [34]. It is worth noting that the observations in petunia concerned IAA content; however, the expression of genes involved in auxin biosynthesis can usually be detected as early as 10–25 min after stress application [81].

**Abscisic acid (ABA)**

ABA is one of the key hormones involved in plant responses to environmental stress, including mechanical stimuli [69,75,82]. Touch stress applied to Phaseolus vulgaris increased ABA levels and inhibited growth [83]. Morphological and physiological changes associated with elevated ABA levels were also observed in studies conducted by Whitehead (1962) and by Weyers and Hillman [84,85]. Recent research has highlighted the complex interplay between ABA and other phytohormones or signaling molecules in conferring stress tolerance in plants. The significance of ABA in mitigating abiotic stress was reviewed by Singh et al. (2023), who underscored its crucial role in enhancing plant growth robustness under various stress conditions [86]. Genetic analyses have not provided a clear demarcation between ABA-dependent and ABA-independent pathways of plant stress responses.

Calcium, which acts as a secondary messenger under multiple stress conditions, represents a strong candidate for mediating the crosstalk between ABA gene expression, ABA accumulation, and the activation of catabolic enzymes responsible for ABA degradation [87]. ABA homeostasis in plants is primarily regulated by its biosynthesis, transport, catabolism, and conjugation with other metabolites, which may occur in a synergistic or antagonistic manner [87].

Abscisic acid and gibberellins represent a classical pair of hormones that function antagonistically in regulating several plant developmental processes, as well as in response to stress [88,89,90]. In contrast to gibberellins, auxin acts synergistically with ABA to regulate key physiological processes during abiotic stress [91]. ABA modulates the auxin signaling pathway mainly by regulating the expression of auxin response factors such as *ARF5*, *ARF6*, and *ARF10* [92]. According to [93,94], elevated ABA levels under stress conditions repress the expression of genes encoding auxin transporters, thereby limiting auxin translocation. Conversely, under homeostatic conditions, reduced ABA levels facilitate auxin transport and subsequently activate the auxin signaling pathway. Similar to ABA, ethylene is also regarded as a classical stress-related phytohormone. Both positive and negative interactions occur between ABA and ethylene, depending on the developmental context. ABA influences ethylene biosynthesis by modulating the expression of ethylene biosynthetic genes, including Ethylene Response Factor 11 and Acyl-CoA Synthetase.

**Gibberellins, Jasmonic Acid, Ethylene**

Gibberellins (GAs), together with auxins, are potent regulators of plant growth, and endogenous levels of these hormones quantitatively determine shoot elongation [86]. Based on reports describing the role of GAs in plant height regulation [95,96], gibberellins strongly influence stem elongation processes. According to [97], gibberellins control numerous aspects of growth and development in higher plants, including seed germination, hypocotyl elongation, stem and reproductive growth, organ and seed development, as well as circadian and light-regulated processes. It is also well established [69,95] that GAs modulate cellulose biosynthesis, thereby influencing cell wall formation. Gibberellins promote cellulose synthesis by activating secondary cell wall protein regulators through DELLA protein inhibition, which simultaneously enhances lignin accumulation [69,96]. In studies conducted on Arabidopsis, sunflower, and kidney bean [98,99,100,101], mechanical stress induced the expression of GIBBERELLIN 2-OXIDASE 6 (GA2ox6) and GA2ox7 genes, whose encoded enzymes catalyze the catabolism of bioactive GA forms, resulting in the inhibition of GA biosynthesis. Reports by [102,103] described the interplay between the jasmonic acid (JA) and gibberellin (GA) pathways in the context of touch stress. It was observed that JA activates GA degradation, thereby reducing plant growth and delaying flowering. According to Kuźma et al. [103], in *Petunia* × *hybrida* “Pegasus Special Burgundy Bicolor”, mechanically stressed plants initiated flowering earlier and produced more flowers than unstressed controls. This phenomenon was attributed both to species-specific and cultivar-dependent characteristics. The early flowering observed under touch stress was explained by increased gibberellin levels during the early stages of stress, which promoted floral induction. However, prolonged exposure to mechanical stress led to a substantial decline in GA levels, resulting in delayed and reduced flowering in severely stressed plants.

Jasmonic acid (JA) integrates multiple hormonal pathways by modulating various cellular processes associated with mechanical stress [18]. It also strongly influences the synthesis of gibberellins (GAs), as mentioned above. According to [75,104,105,106], JA acts antagonistically to ethylene (ET); however, both hormones independently affect the thigmomorphogenetic response [6,64]. Several studies have demonstrated that JA accumulates in the internodes of Bryonia dioica, Medicago truncatula, and Phaseolus vulgaris following mechanical stimulation [20,107]. These findings were further confirmed in Arabidopsis thaliana by Chehab et al. [18]. Mechanical stress also induces the production of ethylene (ET), making it one of the primary regulators of plant responses to mechanical stimuli [75,108]. In Phaseolus vulgaris, the highest ET concentration was observed two hours after exposure to touch stress [109]. The role of ET in the mechanical stress response has been linked to the regulation of stem secondary growth [73,110,111]. According to Erner et al. [75], ethylene may function as a novel repressor of thigmomorphogenesis. The ET pathway also serves as a crucial negative regulator of this process by modulating GA_4_ levels and reducing the expression of the gibberellin catabolic gene GA2ox8 [112].

It is evident that the jasmonic acid and ethylene (ET) pathways can function either synergistically or antagonistically, depending on the physiological context. For instance, they act synergistically in regulating plant immunity against necrotrophic fungi [113,114] and in root hair development [115]. However, under mechanical stress, JA and ET signaling act antagonistically in the regulation of thigmomorphogenesis. Erner et al. [75] proposed that JA and ET independently regulate touch-induced GA_4_ accumulation, exerting opposite effects. They further postulated that the JA pathway promotes thigmomorphogenesis, enabling plants to adapt to adverse environments, whereas the ET pathway prevents plants from overreacting to mechanical stimuli.

### 3.2. Enzymes Induced to Thigmomorphogenesis

During mechanical stress, shoot elongation may be inhibited due to increased activity of indole-3-acetic acid oxidase (IAA oxidase), phenylalanine ammonia-lyase (PAL), cinnamyl alcohol dehydrogenase (CAD), and peroxidases (POD), which are associated with plant defense responses [27,45] (Figure 2). Both PAL and CAD are key enzymes involved in lignin biosynthesis, while POD participates in lignification and suberization of cell walls, leading to reduced cell elongation [109,116,117,118,119,120,121,122,123]. Increased activity of these enzymes, along with the formation of lignified tissues, is considered a nonspecific defense response of plants to mechanical stimuli [27]. Mechanical stimulation also enhances the activity of 1-aminocyclopropane-1-carboxylate (ACC) synthase, a key enzyme in the ethylene biosynthesis pathway [120]. Touch stress has been shown to affect IAA oxidase (IAAO) activity [107,121,122]. This enzyme is typically involved in auxin catabolism and shows a negative correlation with endogenous IAA content [111,124,125]. According to Kuźma et al. [103], in petunia, mechanical stress caused significant alterations in both IAA content and IAAO activity, with a strong negative correlation observed between these two parameters [103]. Studies performed on several *Petunia* × *hybrida* cultivars revealed similar results. In research conducted by Jędrzejuk et al. [27], in the cultivar ‘Pegasus Velvet Picotee’, IAA oxidase activity was higher in brushed plants compared with control plants during the period of touch stress exposure [27]. This effect, however, was not observed in the cultivar ‘Dark Red’. In previous experiments by Jędrzejuk et al. [27] on petunia, increased peroxidase activity during mechanical stimulation (MS) was recorded, which not only contributed to the suppression of IAA synthesis but also to the lignification of cell walls. Although other free radical scavengers in petunia were not analyzed under mechanical stress, it can be inferred from the literature that the plants actively protected themselves against oxidative stress [103,126,127,128,129,130,131]. The peroxidase-mediated oxidative decarboxylation process plays a crucial role in the reduction in auxin levels in plant stems [130,132]. Results from studies conducted in 2020 on petunia also demonstrated higher peroxidase activity in mechanically stressed plants compared to controls [27]. Plants naturally defend themselves against mechanical injury through the production of lignins and suberins in stem tissues [133]. In tomato plants, peroxidase activity significantly increased in the rubbed internodes following the application of mechanical stress [78]. According to Potocka et al. (2018), mechanical stimulation appears to exert stronger effects on root growth than on shoot growth, which has been the primary focus of most studies [64,134,135]. Similar effects were observed in petunia [13,27].

## 4. Plant Response at the Molecular Level—TouCH Gene Expression

Braam and Davis (1990) were the first to report the presence of touch-related genes in plants [36] (Figure 2). In Arabidopsis, *TCH1* encodes a *calmodulin 2* gene (*CaM2*) [30,136], while *TCH2* and *TCH3* encode calmodulin-like (CML) proteins (CML24 and *CML12*) [30,36,136,137,138,139]. In contrast, *TCH4* encodes a xyloglucan endotransglucosylase/hydrolase involved in cell wall modification and remodeling [138,140,141]. Additional genes associated with the touch response include those related to ACC synthase activity [142,143] and protein kinases [144,145].

Expression of *TCH* genes is induced not only by mechanical stimulation but also by environmental factors such as darkness, extreme temperatures, and several growth-promoting or growth-inhibiting hormones [14,138,146,147,148]. The *TCH* genes display distinct developmental expression patterns, often localized in tissues characterized by rapid growth or mechanical strain. For example, *TCH* reporter transgenes were expressed, and TCH proteins accumulated, in the root–shoot junction, elongating hypocotyls, and roots [139,146,147,148]. Regulation of *TCH* gene expression may thus occur both in response to external stimuli and as part of intrinsic morphogenetic pathways [138,139,140]. These developmental expression data support the hypothesis that transient turgor changes at the cellular level may trigger *TCH* gene regulation. It is also possible that plant responses to mechanical stimuli are linked to fundamental processes such as cell expansion [16]. The regulatory properties of TCH proteins may therefore enable physiological and morphological adaptation to environmental cues [138,139].

Mechanical stimulation can also modify plant architecture. In Arabidopsis leaves, touch stress significantly upregulated 1-aminocyclopropane-1-carboxylate synthase (ACS6) and 1-aminocyclopropane-1-carboxylic acid (ACC) gene expression. ACS6 transcripts appeared approximately 5 min after stimulation, reaching a maximum at around 15 min [139,149,150]. At the root apex, mechanical stress induced increased ACC synthase expression within 2–5 min [151], which gradually declined over the following two hours [109].

Prolonged mechanical stimulation enhances JA accumulation [18,152,153]. A single mechanical stimulus activates several JA biosynthetic genes, including Allene Oxide Cyclase 3 (AOC3), Jasmonate-Induced Oxygenase 4 (JAO4), Lipoxygenase 4 (LOX4), and 12-Oxophytodienoate Reductase 3 (OPR3) [20,138]. Expression of these genes is induced within 30 min after stress onset and correlates with elevated JA levels (up to a fourfold increase) for approximately three hours post-stimulation [138]. In wheat, one hour after mechanical stress, the expression of LOX genes was proposed to contribute to enhanced jasmonate precursor synthesis [40,101].

**Figure 3:**
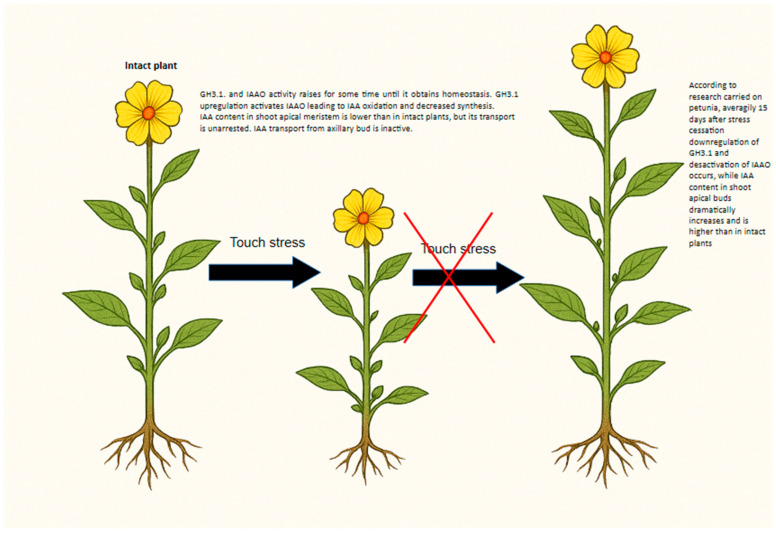
Auxin Homeostasis Regulation Model Proposed under Mechanical Stress.

Several auxin-responsive genes are also differentially regulated in response to mechanical stress, with some (e.g., *GH3.5*, *TAA1*) being downregulated and others (e.g., *YUC5*) upregulated, while overall IAA levels remain relatively stable [109]. In the Arabidopsis root apex, auxin-responsive genes (*SAUR*, *IAA*) were expressed as early as two minutes after exposure to gravity or mechanical stimuli [151]. In *Petunia* × *atkinsiana*, the expression of several TCH genes showed no significant correlation with endogenous auxin synthesis or transport in response to touch stress, whereas calmodulin gene expression largely paralleled that of *GH3.1* at the early stages of stimulation [103] (Figure 3). Expression patterns were also dependent on stress intensity and duration. Plants subjected to 80 or 160 touches per day exhibited differential expression levels, with the 160-touch treatment showing higher expression of *CaM* and *GH3.1* genes compared to plants exposed to 80 touches per day. In petunia, alongside IAAO activity, the GH3.1 gene—which encodes an amido synthetase involved in auxin conjugation—also responded to mechanical stress. *GH3* expression has been associated with enhanced stress tolerance, particularly under drought, low temperature, or salinity conditions [154]. The GH3 enzyme family mediates plant stress responses by regulating auxin homeostasis through the conjugation of free IAA with amino acids, leading to auxin inactivation [154].

According to Kuźma et al. (2025), touch stress increased *GH3.1* expression in petunia, showing a negative correlation between *GH3.1* transcript levels, IAA content, and shoot growth [103] (Figure 3). Conversely, a positive correlation was found between *GH3.1* expression and IAAO activity. Continuing the work of [103] elevated GH3 enzyme production—responsible for IAA conjugation—was strongly associated with growth inhibition and increased stress resistance under mechanical stress. This suggests that the combined increase in auxin oxidation and conjugation functions as a feedback mechanism that modulates auxin levels during prolonged stress, ultimately contributing to growth suppression.

Calmodulins, as mentioned above, act as calcium sensors and mediators, playing essential roles in regulating cellular processes under stress conditions [155]. In petunia [103], all three CaM genes (*CaM53*, *CaM72*, and *CaM81*) exhibited increased expression up to day 56 of mechanical stress, indicating that plants experienced significant physiological strain regardless of stimulation intensity. A strong correlation was observed between *GH3.1* and specific *CaM* genes, depending on the degree of mechanical stress. The variation in *CaM–GH3.1* correlations suggests that distinct calmodulin isoforms may be activated in response to different stress intensities. This may indicate a hierarchical response mechanism, in which *CaM72* is sufficient to regulate auxin homeostasis under moderate stress, whereas stronger stress conditions require the activation of *CaM53* and *CaM81* to maintain auxin balance and prevent excessive growth inhibition.

Mechanical stress also induces expression of GIBBERELLIN 2-OXIDASE 6 (GA2ox6) and GA2ox7, whose enzymes catabolize bioactive GA forms in mechanically stressed tissues of sunflower, kidney bean seedlings, and Arabidopsis [69,100,156]. Conversely, JA antagonizes GA biosynthesis and/or enhances GA degradation to inhibit stem elongation in Nicotiana attenuata [100]. In Arabidopsis, mutants that overaccumulate JA exhibit stunted stems and suppressed growth phenotypes [18]. Loss-of-function in central GA regulators, the DELLA proteins, also impairs JA-responsive gene expression [108]. Therefore, JA is considered a key player in regulating thigmomorphogenesis. A balance between GA catabolism and JA biosynthesis becomes activated during prolonged mechanical stress, transitioning plants from normal growth toward reduced growth and thigmomorphogenetic adaptation. Transcriptomic analyses have revealed that approximately 2.5% of Arabidopsis genes are activated upon mechanical stimulation [157,158]. In poplar, a single touch affects the expression of about 6% of genes within the first two hours following stimulation [159]. Although plant responses to mechanical stimuli are complex and involve multiple signaling pathways, the complete molecular network remains only partially characterized. It can be hypothesized that several interdependent regulatory pathways, rather than a single primary mechanism, are required for the coordination of TCH gene expression under mechanical stress.

## 5. Thigmomorphogenesis Mechanism in Monocots

### 5.1. Stem Reaction to Mechanical Stress in Monocots

Mechanical stimulation in cereals—particularly during the seedling stage—has been known for thousands of years. This traditional process, called mugifumi, involves treading on young plants and results in more resilient individuals with higher yields compared to untreated controls [2,160]. According to earlier studies [80,161,162,163,164,165,166], monocots respond to mechanical stress in a manner similar to dicots, including reduced elongation and activation of various regulatory networks such as hormones, proteins, transcription factors, and target genes. These responses manifest through alterations in physiology, morphology, and biomechanical properties. The primary differences, however, are observed at the cellular and vascular levels.

In monocots, mechanical stress applied to the stem often results in breakage occurring below the height of the inflorescence [167]. Younger internodes are the most sensitive and adaptive to mechanical stimulation, which typically leads to a decrease in their length [160,168]. Shorter internodes are more resistant to external forces and less prone to tissue damage; for instance, field-grown lodging-resistant sorghum plants exhibited lower flexural stiffness but stronger stems overall. Mechanical stimulation in monocots reduces internode elongation and induces distinct anatomical changes within the stem [169].

### 5.2. Differences in Secondary Cell Wall Structure Between Eudicots and Monocotyledons

The main function of secondary cell walls is to provide mechanical strength to vascular and structural tissues. They are composed primarily of cellulose, hemicellulose, and lignin. However, in monocotyledons, secondary cell walls differ from those of eudicots in both the chemical composition of hemicelluloses and the pathways of lignin biosynthesis. Grasses, unlike eudicots, are capable of producing mixed-linkage glucans (MLGs)—a type of wall polysaccharide that is rarely found in eudicot species [170]. Another key difference in stem structure between monocots and eudicots lies in the absence of a vascular cambium and lateral meristems in monocots. Instead, stem elongation in grasses is achieved through the division and elongation of cells within intercalary meristems [157].

### 5.3. Differences in Specific Gene Expression Between Eudicots and Monocotyledons

Expression of touch-related (TCH) genes represents a molecular hallmark of plant responses to mechanical stress. It is well documented that touch induces substantial transcriptional changes, affecting approximately 2.5–10% of genes in eudicots such as Arabidopsis thaliana and poplar [119,159,171]. In monocots such as sorghum, the response to touch appears slower, likely due to the presence of the leaf sheath, which imposes mechanical constraints on developing inflorescences and thereby dampens gene expression changes. When the sheath was removed, however, 42% of genes were differentially expressed within a 9-h period [172]. Recent studies on cereal species, including oat, wheat, and barley, have shown that 2–5% of TCH genes are differentially expressed within two hours of mechanical stimulation in young leaves [172]. In grasses, cell wall–related genes are also actively involved in mechanostimulation responses. Mechanical stress upregulates genes associated with glucan-based cell wall synthesis, whereas genes involved in lignin regulation tend to be downregulated.

In summary, the response of grasses to mechanical stress differs from that of eudicots primarily at the cellular level. These differences include the unique chemical structure of secondary cell walls, the absence of a vascular cambium and lateral buds, and the involvement of cell wall–related genes in the mechanostimulation response process.

## 6. Conclusions

Mechanical stress induces a wide range of changes in plants, beginning with the activation of gene expression (e.g., TCH genes), and continuing through biochemical (activation of various enzymes), physiological, and morphological responses. Although mechanical stress has been recognized since the 1970s as thigmomorphogenesis, its mechanisms of action and plant responses to this stressor remain unclear. This review highlights recent advances in the research conducted on petunia, revealing that auxin biosynthesis is significantly inhibited only after 30 days of continuous mechanical stimulation, while auxin transport remains unaffected. The work also references studies indicating a strong correlation between the expression of selected calmodulins and the GH3.1 gene.
